# Advancements and Future Directions in Polycythemia Vera Research: A Bibliometric Analysis

**DOI:** 10.7759/cureus.61774

**Published:** 2024-06-06

**Authors:** Bibin Xavier

**Affiliations:** 1 Marian Institute of Management, Marian College Kuttikkanam (Autonomous), Kuttikkanam, IND

**Keywords:** hematology, vos viewer, biblioshiny, bibliometric analysis, polycythemia vera

## Abstract

This research provides a comprehensive bibliometric analysis of polycythemia vera (PV) research trends, encompassing data from 1969 to 2024. Utilizing advanced tools, key findings reveal a notable increase in scientific production over time, reflecting growing interest and investment in PV research. Prominent themes include genetic studies, targeted therapies, and precision medicine approaches. The analysis identifies leading authors, institutions, and countries contributing to PV research, highlighting the importance of global collaboration. The study emphasizes the need to broaden genetic investigations, explore the bone marrow microenvironment, and enhance precision medicine strategies. The implications of this research extend to clinical practice, with potential advancements in diagnostics, treatments, and patient outcomes. Ultimately, addressing these challenges and embracing emerging opportunities can propel PV research forward, fostering innovation and improving the lives of affected individuals.

## Introduction and background

Polycythemia vera (PV), a myeloproliferative neoplasm (MPN) characterized by the overproduction of red blood cells, remains a clinically challenging entity despite advancements in medical science [1]. It is a rare and incurable blood cancer associated with the overproduction of blood cells in the bone marrow, part of a group of related blood cancers known as MPNs, with almost all patients having a mutation in the JAK2 gene [2]. PV is a clinically challenging disorder, as its precise cause remains unknown, and current treatment strategies focus primarily on reducing hematocrit levels and managing symptoms rather than curing the disease [3]. Common treatments include phlebotomy, aspirin, and cytoreductive therapy, but their efficacy in preventing disease transformation, such as progression to leukemia, is unclear [4]. For instance, while phlebotomy helps manage blood thickness, it does not address the underlying genetic abnormalities driving the disease. The JAK2 V617F mutation is a common feature of PV, and preclinical models are being used to explore potential therapeutic targets [5]. Current treatment strategies for PV focus on reducing thrombohemorrhagic risk by controlling blood counts and inhibiting platelets, but often fail to address disease-related symptoms or biologically modify the disease. There is a shift toward a comprehensive treatment strategy that aims to improve quality of life and prevent disease progression [6]. First-line treatments for PV include low-dose aspirin and goal-directed phlebotomy, which aim to reduce the risk of thrombosis and manage hematocrit levels. When additional intervention is required, cytoreductive therapy with hydroxyurea is commonly employed to decrease the production of blood cells. In cases where hydroxyurea is insufficient or not tolerated, second-line agents such as pegylated interferon-alfa, busulfan, and ruxolitinib are utilized to further manage the disease and alleviate symptoms [7]. Despite these advancements, the need for more effective therapies that improve quality of life and prevent disease progression remains critical.

Conducting a bibliometric analysis of PV is crucial given the complexities and evolving nature of this MPN. Despite advancements in medical science, PV remains clinically challenging and incurable, with its management focused primarily on symptom control and reducing hematocrit levels. Understanding research trends, key contributions, and collaborative networks through bibliometric analysis can illuminate shifts in research priorities and highlight influential studies, aiding in the development of more effective treatment strategies. Given that PV is associated with a mutation in the JAK2 gene and current treatments often fail to biologically modify the disease, identifying emerging areas of interest and gaps in the literature is essential for guiding future research. Furthermore, the global distribution of research efforts can be assessed, identifying regions leading advancements and those requiring more focus. This analysis not only informs clinical practice by identifying effective treatments and management strategies but also supports policymakers and funding bodies in allocating resources to high-impact research areas. In short, this bibliometric analysis provides a comprehensive overview of the PV research landscape, fostering informed and strategic advancements in understanding and treating this complex disease.

This research paper endeavors to conduct a meticulous bibliometric analysis of the corpus of PV-related literature housed within the PubMed database. PubMed stands as a cornerstone resource in the realm of biomedical research, housing an extensive collection of peer-reviewed articles, reviews, and clinical studies. Leveraging the wealth of information contained within this repository, our study seeks to illuminate the trajectory of PV research, identifying seminal publications, prolific authors, and pivotal themes that have shaped the discourse surrounding this condition. Through the lens of bibliometrics, we aim to provide a panoramic view of the scientific landscape surrounding PV, delineating the growth patterns, collaborative networks, and knowledge dissemination channels prevalent within the research community. By scrutinizing publication trends over time, we endeavor to discern shifting priorities and emerging focal points, thus aiding in the formulation of informed strategies for future research endeavors and clinical interventions. Moreover, by elucidating the geographic distribution of research output and the prevalence of interdisciplinary collaborations, our analysis seeks to underscore the global impact of PV research and the interdisciplinary nature of efforts aimed at unraveling its complexities. By distilling the collective wisdom embedded within the scholarly literature, our study aims to empower stakeholders, ranging from clinicians and researchers to policymakers and patients, with actionable insights that can inform decision-making processes and facilitate the advancement of therapeutic strategies.

Methodology

**Figure 1:**
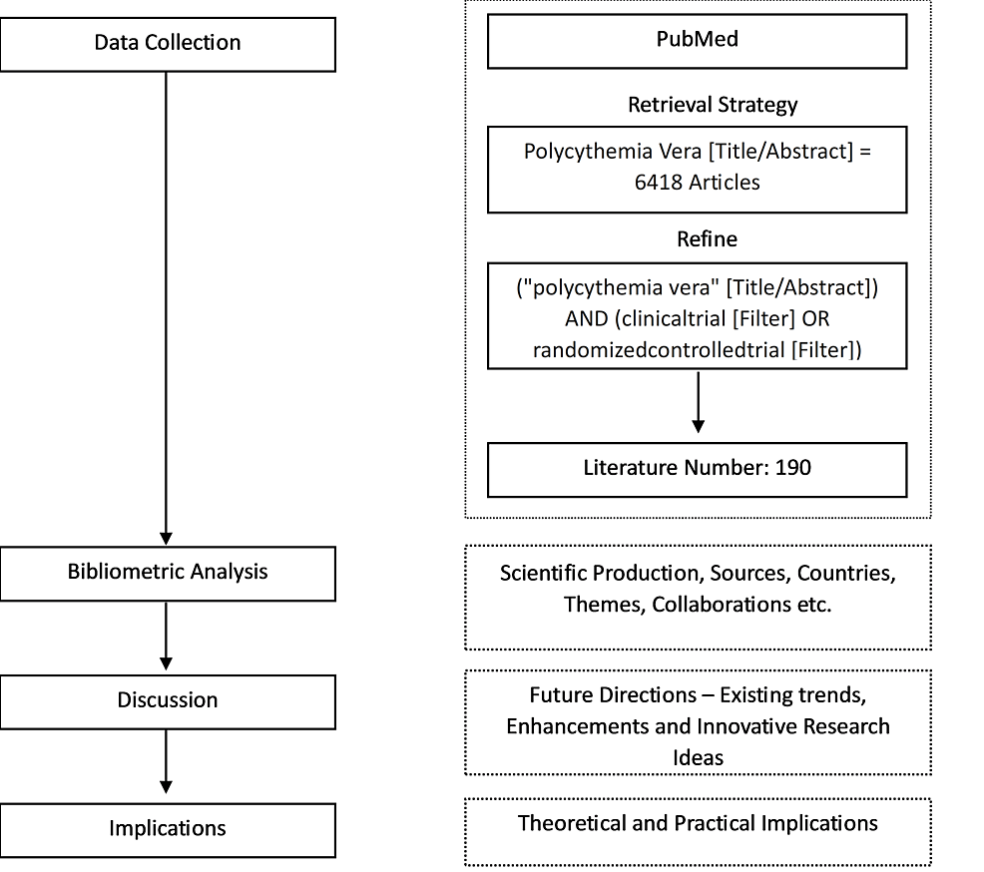
Analytical procedure of the bibliometric analysis

The research involved a systematic approach (Preferred Reporting Items for Systematic reviews and Meta-Analyses (PRISMA)) (Figure 1), beginning with data collection from the PubMed database using the search query “polycythemia vera” [Title/Abstract], which retrieved 6,418 articles. To ensure the relevance and quality of the analyzed literature, this was refined further to 190 articles by applying filters to limit the search to clinical trials and randomized controlled trials. This filtering logic was based on the need to focus on high-quality, evidence-based research that directly impacts clinical practice and patient outcomes. The bibliometric analysis focused on examining scientific production over time, identifying key sources, geographic distribution, prevalent themes, and collaborative networks among authors and institutions. The findings were discussed in terms of current research trends, significant contributions, and identified gaps. The study outlined both theoretical and practical implications, emphasizing contributions to scientific understanding and impacts on clinical practice. Finally, future research directions were proposed, suggesting enhancements in methodologies and innovative approaches to address existing challenges in PV research.

## Review

Bibliometric analysis

For the bibliometric analysis, we employed Biblioshiny and VOSviewer, two powerful software tools renowned for their effectiveness in handling bibliometric data and facilitating network analysis. Biblioshiny offers a user-friendly interface for managing bibliographic data and conducting comprehensive bibliometric analyses, while VOSviewer excels in visualizing and analyzing complex bibliometric networks. Utilizing these advanced tools, we extracted valuable insights from the literature on social accounting. Our in-depth analyses identified trends, patterns, and relationships among various entities, such as authors, publications, and keywords. The visualizations generated by VOSviewer allowed us to explore the intricate network of scholarly connections within the field, providing a comprehensive overview of the research landscape.

Characteristics of Literature

As shown in Table 1, the bibliometric analysis covered data from 1969 to 2024 across 69 journals and books, with 190 documents, an annual growth rate of 2.02%, and an average document age of 15 years. Collaboration was notable, involving 1,420 authors with an average of 11.2 co-authors per document and 15.26% of documents featuring international co-authorships.

**Table 1:** Characteristics of the literature

Description	Results
Main information about the data	
Time span	1969:2024
Sources (journals, books, etc.)	69
Documents	190
Annual growth rate %	2.02
Document average age	15
Document contents	
Keywords Plus (ID)	1,011
Author’s keywords (DE)	1,011
Authors	
Authors	1,420
Authors of single-authored documents	7
Authors collaboration	
Single-authored documents	9
Co-authors per document	11.2
International co-authorships %	15.26

Annual Scientific Production

**Figure 2:**
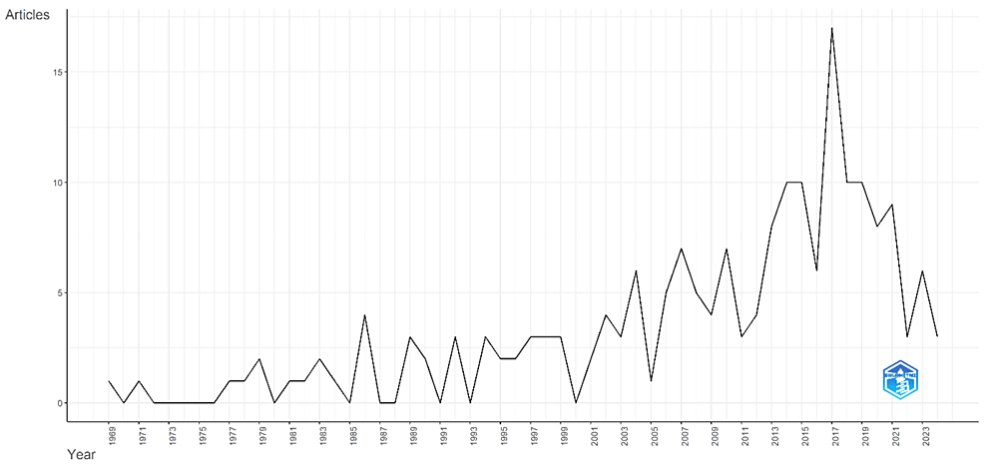
Annual scientific production This figure illustrates the annual scientific production of articles on PV from 1969 to 2024. The x-axis represents the years, while the y-axis indicates the number of articles published each year. The trend demonstrates a variable but generally increasing pattern of research activity over this period. PV, polycythemia vera

The annual scientific production on PV (Figure 2) demonstrates a variable but generally increasing trend over the decades from 1969 to 2024. Initial research output was sparse, with only one article published in 1969 and intermittent publications in the following years. Notable increases began in the late 1980s and early 1990s, with a peak of four articles in 1986 and a steady rise in the 1990s, reaching up to three articles per year. The early 2000s saw a significant boost in scientific production, with six articles in 2004 and a noticeable increase to seven articles in 2007 and 2010. This upward trend continued, culminating in a substantial surge to 17 articles in 2017. The following years maintained a higher level of research activity, with a peak of 10 articles annually from 2014 to 2019, a slight decline but consistent output with eight articles in 2020, nine in 2021, and a tapering to three articles by 2024. This trajectory highlights the growing interest and research investment in PV over time, reflecting advancements in understanding and treating this condition.

Most Relevant Sources

As shown in Table 2, the most prolific source for PV research is Blood, publishing 28 articles and underscoring its central role in hematology. Other significant contributors include Annals of Hematology with 19 articles, Haematologica and the International Journal of Hematology each with 11 articles, and Leukemia Research with 10 articles, indicating their substantial impact in the field. The highly prestigious New England Journal of Medicine contributed seven articles, showcasing its influence on clinical research. Collectively, these journals highlight the interdisciplinary and international efforts in advancing the understanding and treatment of PV.

**Table 2:** Most relevant journals This table lists the journals that have published the most articles on PV, highlighting the key sources of research in this field. PV, polycythemia vera

Sources	Articles
Blood	28
Annals of Hematology	19
Haematologica	11
International Journal of Hematology	11
Leukemia Research	10
American Journal of Hematology	8
The New England Journal of Medicine	7
Gan to Kagaku Ryoho. Cancer & Chemotherapy	6
Leukemia	6
European Journal of Haematology	5
Journal of Clinical Oncology: Official Journal of the American Society of Clinical Oncology	5
Cancer	4

Core Sources by Bradford’s Law

**Figure 3:**
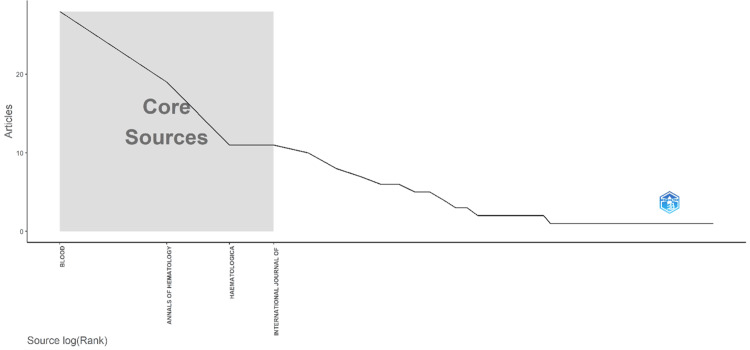
Core sources by Bradford’s law The legend for core sources by Bradford’s law depicts the relationship between the number of articles published (y-axis) and the logarithmic rank of sources (x-axis). The y-axis signifies the volume of scholarly work contributed by different sources, while the x-axis quantifies the ranking of these sources on a logarithmic scale. Core sources, highlighted within the gray square on the graph, denote the pivotal contributors that exert significant influence within the scholarly landscape, as identified by Bradford’s law.

Applying Bradford’s law to the bibliometric analysis of PV research (Figure 3, Table 3), core journals in the field were identified across three zones based on publication frequency. Zone 1, encompassing the most prolific sources, includes Blood with 28 articles, Annals of Hematology with 19 articles, and Haematologica and the International Journal of Hematology, each with 11 articles. Zone 2 comprises sources with moderate publication frequency, such as Leukemia Research, American Journal of Hematology, and The New England Journal of Medicine, among others. Zone 3 encompasses sources with the least frequency of publications. This classification underscores the significance of Zone 1 journals as primary sources for PV research, with Zone 2 and Zone 3 journals providing supplementary insights into the field.

**Table 3:** Core sources by Bradford’s law The legend for the table provides a comprehensive overview of the academic journals listed, their respective rankings based on publication frequency, and additional statistical metrics. “Journal” denotes the titles of the journals, while “Rank” showcases their position in terms of publication frequency. “Freq” highlights the number of articles published by each journal, with “cumFreq” representing the cumulative frequency, reflecting the total articles published up to the respective journal. Lastly, “Zone” categorizes the journals into different zones based on their cumulative frequency, with Zone 1 signifying the most prolific journals and subsequent zones indicating decreasing publication frequency.

Journal	Rank	Freq	cumFreq	Zone
Blood	1	28	28	Zone 1
Annals of Hematology	2	19	47	Zone 1
Haematologica	3	11	58	Zone 1
International Journal of Hematology	4	11	69	Zone 1
Leukemia Research	5	10	79	Zone 2
American Journal of Hematology	6	8	87	Zone 2
The New England Journal of Medicine	7	7	94	Zone 2
Gan to Kagaku Ryoho. Cancer & Chemotherapy	8	6	100	Zone 2
Leukemia	9	6	106	Zone 2
European Journal of Haematology	10	5	111	Zone 2
Journal of Clinical Oncology: Official Journal of the American Society of Clinical Oncology	11	5	116	Zone 2
Cancer	12	4	120	Zone 2
Blood Cancer Journal	13	3	123	Zone 2
Experimental Hematology	14	3	126	Zone 2
Annales De Medecine Interne	15	2	128	Zone 2
Blood Advances	16	2	130	Zone 3
Cancer Treatment Reports	17	2	132	Zone 3
Clinical Lymphoma, Myeloma & Leukemia	18	2	134	Zone 3
Hematology (Amsterdam, Netherlands)	19	2	136	Zone 3
Leukemia & Lymphoma	20	2	138	Zone 3
Medical Oncology (Northwood, London, England)	21	2	140	Zone 3
Orvosi Hetilap	22	2	142	Zone 3
Seminars in Hematology	23	2	144	Zone 3
Acta Dermato-Venereologica	24	1	145	Zone 3
Acta Haematologica	25	1	146	Zone 3

Most Relevant Authors

In the analysis of authors contributing to research on PV (Table 4), several key figures emerged, with varying degrees of influence. Among them, Vannucchi AM stands out with 24 articles, representing a substantial contribution to the field. Verstovsek S follows closely with 23 articles, while Kiladjian JJ and Mesa RA have contributed 16 and 12 articles, respectively. Other notable authors include Griesshammer M, Hoffman R, and Rambaldi A, each with 11 articles. Barbui T and Harrison CN have authored 10 articles each, while Mesa R and Passamonti F have contributed 10 and nine articles, respectively. Rain JD and Najean Y have a higher fractionalized contribution, suggesting a significant impact despite fewer articles authored.

**Table 4:** Most relevant authors “Authors” lists the names of individuals who have authored scholarly articles, while “Articles” denotes the number of articles attributed to each author. The metric “Articles fractionalized” offers a nuanced perspective by presenting the fractional contribution of each author relative to the total output in the field, with higher values indicating a more substantial impact.

Authors	Articles	Articles fractionalized
Vannucchi AM	24	1.38
Verstovsek S	23	2.52
Kiladjian JJ	16	1.64
Mesa RA	12	0.82
Griesshammer M	11	1.13
Guglielmelli P	11	0.75
Hoffman R	11	0.84
Rambaldi A	11	0.44
Barbui T	10	0.5
Harrison CN	10	0.72
Mesa R	10	0.69
Passamonti F	9	0.53
Rain JD	9	2.33
Gisslinger H	8	0.66
Goldberg JD	8	0.82
Kantarjian H	8	0.82
Najean Y	8	3.04
Najfeld V	8	0.55
Tefferi A	8	1.02

Co-Authorship

**Figure 4:**
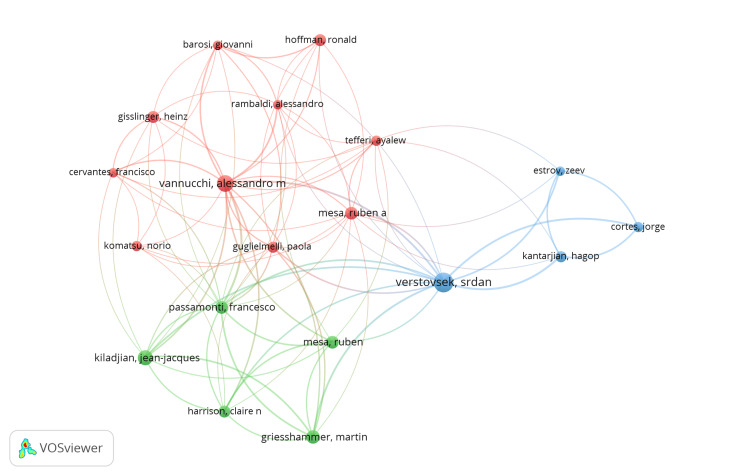
Co-authorship The circle surrounding each author’s name in the network visualization reflects the number of publications they have co-authored on PV. A larger circle indicates a greater number of co-authored articles attributed to the author. The thickness of the connecting lines between authors corresponds to the quantity of co-authored articles shared between them; thicker lines signify a higher number of shared publications. Additionally, authors connected by lines of common color—red, light blue, or light green—have collaborated on articles together, indicating shared research efforts. PV, polycythemia vera

The map depicts co-authorship patterns in polycythemia research (Figure 4). Nodes represent individual researchers, with their size proportional to the number of publications on polycythemia (full-count authorship likely). Links connecting the nodes indicate co-authorship, with thicker and darker lines signifying a greater number of co-authored papers between researchers. It allows for the identification of prominent researchers in the field. For instance, the larger nodes likely correspond to prolific authors such as Srdan Verstovsek, Francesco Passamonti, and Alessandro Vannucchi. Additionally, the network of connections reveals collaborative relationships between researchers. The presence of numerous links suggests a significant degree of collaboration within the field of polycythemia research.

Most Relevant Affiliations

Tisch Cancer Institute emerges as a leading contributor with 49 articles, followed closely by the University of Florence with 43 articles and Anderson Cancer Center with 33 articles. Zealand University Hospital, Mount Sinai School of Medicine, University of Texas M., and Mayo Clinic also demonstrate substantial contributions with 29, 24, 22, and 21 articles, respectively. Noteworthy institutions such as Cleveland Clinic Taussig Cancer Institute, Juntendo University Graduate School of Medicine, and All India Institute of Medical Sciences have each contributed 20 articles. Lee Moffitt Cancer Center, Stanford University School of Medicine, and University of Michigan Health, among others, have also made significant contributions, with 19 articles each (Table 5).

**Table 5:** Most relevant affiliations The most relevant affiliations provide a succinct overview of institutions or organizations associated with scholarly articles. “Affiliation” lists the names of these entities, while “Articles” quantifies the number of scholarly articles attributed to each affiliation, reflecting their research output and contribution to the academic landscape.

Affiliation	Articles
Tisch Cancer Institute	49
University of Florence	43
Anderson Cancer Center	33
Zealand University Hospital	29
Mount Sinai School of Medicine	24
University of Texas M.	22
Mayo Clinic	21
Cleveland Clinic Taussig Cancer Institute	20
Juntendo University Graduate School of Medicine	20
All India Institute of Medical Sciences	19
Lee Moffitt Cancer Center	19
Stanford University School of Medicine	19
The Mayo Clinic	19
University of Michigan Health	19
Weill Cornell Medical College	19
Rwth Aachen University	18
London Health Sciences Centre	17
Medical University of Vienna	14
Harvard Medical School	13
University of Texas MD Anderson Cancer Center	13
Western University	13
Azienda Ospedaliera Universitaria Careggi	12

Country Scientific Production

**Figure 5:**
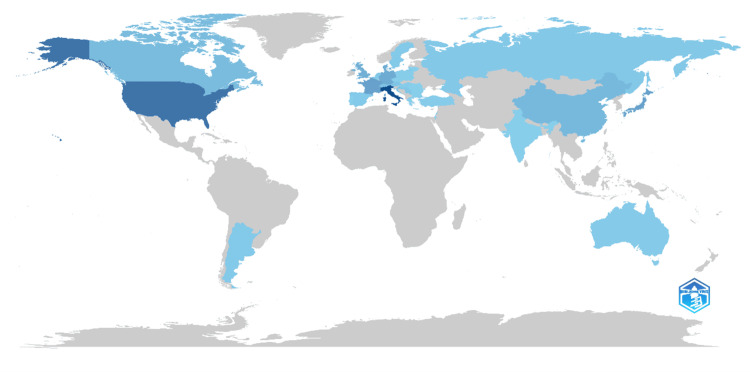
Country scientific production This world map visualizes the distribution of scientific articles on PV, published between 1969 and 2024. Countries are color-coded from dark blue (highest production) to light blue (lowest production), indicating their contribution levels. PV, polycythemia vera

The scientific production on PV is distributed across various countries, with Italy leading the way with 288 articles, followed by the USA with 196 articles (Figure 5). Japan and France are also significant contributors, with 94 and 93 articles, respectively, while Denmark and Germany have each produced 75 articles. The UK follows closely with 65 articles, while China and Canada have contributed 45 and 38 articles, respectively. Switzerland and Austria have produced 24 and 20 articles, while Croatia and Serbia have each contributed 20 articles. Other notable contributors include the Netherlands and Russia with 13 articles each, and Argentina, Spain, and Sweden with 11 articles each. Australia and Belgium have produced seven articles each, while Hungary, Turkey, Poland, Greece, and Israel have each contributed fewer articles, ranging from two to six. This distribution underscores the global collaboration and diverse research efforts in advancing knowledge on PV.

Trend Topics

**Figure 6:**
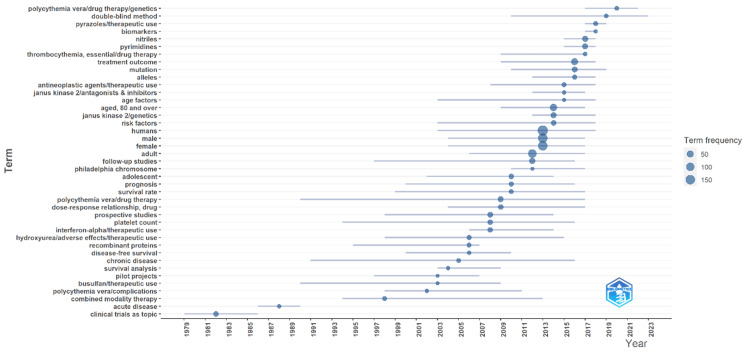
Trend topics This figure presents an analysis of the trend topics related to PV from 1979 to 2023. The circle represents the year in which that topic was most popular, with the size of the circle corresponding to the frequency of the term's usage in published articles. The smaller circles indicate less frequent usage, while larger circles denote higher frequency. The lines through the circles indicate the span of years during which each topic was prevalent in the literature. PV, polycythemia vera

Recent topics in PV research highlight contemporary trends and emerging areas of focus within the field (Figure 6, Table 6). Since 2015, there has been an increasing interest in nitriles and pyrimidines, which were frequently studied from 2015 to 2018. The genetic aspects of PV have also gained significant attention, particularly the JAK2 gene, with research on JAK2 genetics peaking from 2012 to 2018 and studies on JAK2 antagonists and inhibitors between 2012 and 2017. The double-blind method has become more prevalent in recent clinical trials, reflecting a rigorous approach to validating new treatments from 2010 to 2023. Topics such as pyrazole therapeutic use and the identification and application of biomarkers emerged prominently between 2017 and 2019, indicating a focus on new drug development and precision medicine. Additionally, studies on treatment outcomes and mutation analysis have remained critical, with significant research activity from 2016 to 2019. The intersection of PV drug therapy and genetics has seen a notable increase in recent years, particularly from 2017 to 2022, suggesting a trend toward personalized medicine approaches. This recent focus on genetic markers, advanced therapeutic agents, and robust clinical trial methodologies underscores a dynamic and evolving research landscape aimed at improving patient outcomes and understanding the underlying mechanisms of PV.

**Table 6:** Trend topics PV, polycythemia vera

Item	Frequency	First appearance	Median year	Latest observation
Clinical trials as topic	14	1979	1982	1986
Acute disease	6	1986	1988	1990
Combined modality therapy	9	1994	1998	2013
PV/complications	6	1998	2002	2011
Busulfan/therapeutic use	5	1990	2003	2009
Pilot projects	5	1997	2003	2007
Survival analysis	6	2003	2004	2009
Chronic disease	8	1991	2005	2016
Hydroxyurea/adverse effects/therapeutic use	10	1998	2006	2015
Recombinant proteins	9	1995	2006	2007
Disease-free survival	6	2000	2006	2010
Prospective studies	20	1998	2008	2014
Interferon-alpha/therapeutic use	15	2006	2008	2014
Platelet count	15	1994	2008	2016
PV/drug therapy	22	1990	2009	2017
Dose-response relationship, drug	14	2004	2009	2017
Adolescent	14	2002	2010	2014
Prognosis	11	2000	2010	2016
Survival rate	9	1999	2010	2017
Adult	108	2006	2012	2017
Follow-up studies	29	1997	2012	2016
Philadelphia chromosome	5	2010	2012	2017
Humans	190	2003	2013	2018
Female	145	2004	2013	2017
Male	145	2004	2013	2017
Aged, 80 and over	61	2009	2014	2017
JAK2/genetics	27	2012	2014	2018
Risk factors	14	2003	2014	2018
Antineoplastic agents/therapeutic use	9	2008	2015	2018
JAK2/antagonists and inhibitors	6	2012	2015	2017
Age factors	5	2003	2015	2018
Treatment outcome	50	2009	2016	2018
Mutation	20	2010	2016	2019
Alleles	10	2012	2016	2018
Nitriles	26	2015	2017	2018
Pyrimidines	25	2015	2017	2018
Thrombocythemia, essential/drug therapy	7	2009	2017	2017
Pyrazoles/therapeutic use	9	2017	2018	2019
Biomarkers	6	2017	2018	2018
Double-blind method	7	2010	2019	2023
PV/drug therapy/genetics	8	2017	2020	2022

Word Cloud

**Figure 7:**
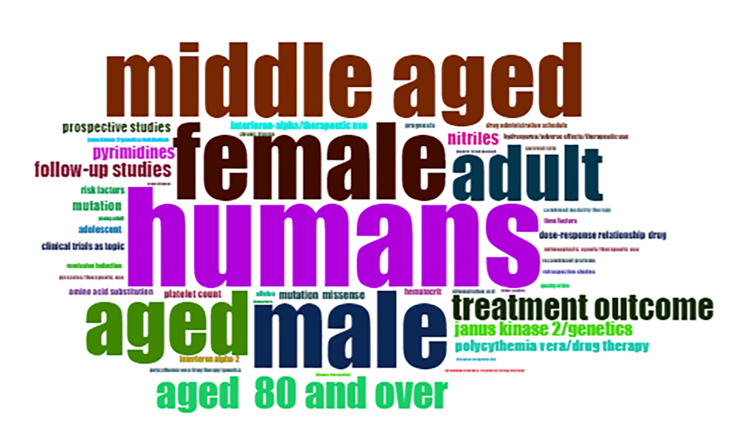
Word cloud The word cloud visually presents words or phrases related to PV, with the size of each word indicating its frequency or significance within the context of the topic. Larger words represent higher frequency or greater importance. PV, polycythemia vera

The word cloud of recent PV research (Figure 7) highlights key terms and their frequencies, reflecting important areas of focus. “Humans” is the most mentioned term with 190 mentions, followed by “female” and “male” at 145 each, indicating the demographic scope of studies. Age-related terms such as “middle-aged” (142 mentions) and “aged” (122 mentions) are also prominent. “Adult” appears 108 times, and “aged 80 and over” is noted 61 times. Significant research themes include “treatment outcome” (50 mentions) and “follow-up studies” (29 mentions), emphasizing the focus on therapeutic efficacy and longitudinal research. Genetic aspects like “Janus kinase 2/genetics” (27 mentions) are critical, along with emerging compounds like “nitriles” (26 mentions) and “pyrimidines” (25 mentions). Key management terms include “polycythemia vera/drug therapy” (22 mentions), “mutation” (20 mentions), and “prospective studies” (20 mentions). Treatment-specific terms such as “interferon-alpha/therapeutic use” and “platelet count” (15 mentions each), “clinical trials as a topic,” “dose-response relationship drug,” and “risk factors” (14 mentions each) are also significant. This word cloud highlights the multifaceted approach to PV research, encompassing genetic, demographic, and therapeutic dimensions.

Thematic Map

**Figure 8:**
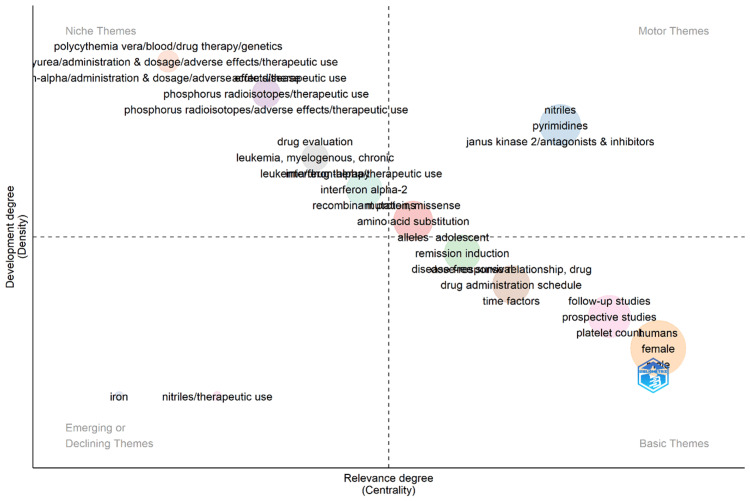
Thematic map Divided into four distinct quadrants—Niche, Motor, Emerging, and Declining—the map categorizes themes or concepts within the field. “Niche” represents specialized topics or concepts within the realm of PV that have a limited mainstream focus but may be of significant interest to specific subsets of researchers or practitioners. “Motor” encompasses central themes or concepts that drive discussions, research, and advancements within the field of PV. These topics are pivotal in shaping the direction and progress of scholarly inquiry. “Emerging or Declining” highlights themes or concepts that are either gaining traction and increasing in relevance over time (Emerging) or diminishing in prominence and becoming less significant (Declining) within the context of PV. “Basic Themes” represents fundamental or foundational topics related to PV that serve as building blocks for further exploration and understanding of the condition. These themes provide essential context and knowledge within the field. PV, polycythemia vera

The thematic map visualizes the results of a bibliometric analysis on PV research, categorizing themes into four distinct areas: niche, motor, emerging, and basic (Figure 8). Niche themes, situated on the periphery of the map, represent less-developed research areas but hold promise for future exploration. Examples include urea, phosphorus radioisotopes, and nitriles. Motor themes, occupying a more central location, signify highly relevant and well-developed areas of research. These themes focus on drug evaluation, JAK2, and leukemia. Emerging or declining themes, positioned between the center and periphery, represent areas of research gaining or losing momentum. Examples include interferon alpha-2, recombinant missense mutations, and amino acid substitution. Finally, basic themes, situated at the map’s core, represent foundational and highly developed research areas. Iron deficiency and platelet count are illustrative examples of basic themes. Thematic map positioning reflects a theme’s relative importance (centrality) and current state of development (density) within the field of PV research. Themes located closer to the map’s center are considered more relevant to the core research questions, while those on the periphery are considered less relevant. Thematic map placement along the vertical axis indicates the relative maturity of the research area. Themes positioned higher up the map represent more developed areas of research, while those lower down represent less developed areas. This thematic map provides a valuable tool for researchers by visually summarizing the current research landscape of PV, highlighting both established and emerging areas of inquiry.

Findings

The annual scientific production of PV has shown a marked increase from 1969 to 2024. While initial research output was limited, there has been a significant surge since the late 1980s, with notable peaks in research activity during the early 2000s and again in 2017. This growth trajectory underscores the increasing recognition of PV’s clinical importance and the advancements in understanding and managing the condition. Key journals such as Blood, Annals of Hematology, Haematologica, and the International Journal of Hematology have emerged as central sources of PV research, reflecting their pivotal role in the field of hematology. Additionally, The New England Journal of Medicine and Journal of Clinical Oncology have made significant contributions, highlighting the interdisciplinary and clinical relevance of PV research.

Prominent contributors to PV research include authors like Vannucchi AM, Verstovsek S, and Kiladjian JJ, who have authored numerous influential articles. Leading institutions such as the Tisch Cancer Institute, University of Florence, and Anderson Cancer Center have been at the forefront of PV research, indicating robust research programs and collaborative efforts in this domain. Geographically, Italy and the USA are the top producers of PV research, followed by Japan, France, and Denmark, illustrating the global nature of PV research and the collaborative efforts across various countries. Recent research trends indicate a significant focus on the genetic aspects of PV, particularly the JAK2 gene, and the development of JAK2 inhibitors. There is also an emphasis on treatment outcomes, clinical trials, and the identification of biomarkers, reflecting a shift toward precision medicine and enhanced therapeutic strategies.

Discussion

The increasing trend in PV research output underscores a heightened scientific and clinical interest in this hematologic disorder over the past several decades. This escalation can be attributed to advancements in molecular biology and genetics, which have elucidated key pathophysiological mechanisms underlying PV, particularly the discovery of the JAK2 V617F mutation [8]. This mutation, present in the majority of PV patients, has catalyzed significant research into targeted therapies, leading to the development of JAK2 inhibitors [9].

Despite these advancements, several critical gaps remain in our understanding of PV, particularly in elucidating its precise etiology and developing curative treatments. The focus on JAK2 mutations has been a pivotal development in PV research, enabling more precise diagnosis and the development of targeted therapies. However, the singular focus on JAK2 mutations [10-13] may overlook other potential genetic and molecular contributors to PV. Understanding the genetic and environmental factors contributing to PV pathogenesis is crucial for developing targeted therapies and improving patient outcomes. Despite advancements in identifying the JAK2 V617F mutation, other genetic and environmental contributors to PV remain understudied. Investigating these factors can provide insights into disease mechanisms and identify new therapeutic targets. Recent studies suggest the involvement of other mutations and epigenetic modifications [14-16], indicating a more complex genetic landscape. This complexity necessitates broader genomic studies to identify additional therapeutic targets and to understand the heterogeneity of the disease. Furthermore, the role of the bone marrow microenvironment and its interaction with PV cells remains underexplored and warrants further investigation [17,18]. Large-scale genomic studies and environmental assessments can be conducted in PV patients and control groups. Advanced sequencing technologies and multi-omics approaches can be employed to identify genetic variants, epigenetic modifications, and environmental exposures associated with PV. The expected outcome is an enhanced understanding of the interplay between genetic predisposition and environmental triggers [19-21] in PV development, leading to the identification of new biomarkers and therapeutic targets for personalized treatment strategies.

International collaboration in PV research is vital, as it allows for the pooling of resources, expertise, and data, enabling large-scale studies that would otherwise be challenging within single institutions or countries. Despite this collaboration, there remains an imbalance in research contributions, with high-income countries such as the USA, Italy, and Japan leading the field. To address this, there is a pressing need to enhance research capacity and output in underrepresented regions, particularly in low- and middle-income countries. This can be achieved through initiatives such as international consortia, capacity-building programs, and equitable distribution of research funding. Strengthening global collaboration in PV research promotes knowledge exchange, resource sharing, and capacity building, ultimately leading to more equitable research outcomes and enhanced diversity in study findings. By establishing international consortia and collaborative networks involving researchers, clinicians, and policymakers from diverse geographic regions and facilitating data sharing, joint research projects, and training programs, the research capacity in underrepresented areas can be significantly enhanced. The expected outcome is an improvement in research capacity and output in low- and middle-income countries, accompanied by enhanced diversity and inclusivity in PV research, resulting in more comprehensive and representative study findings.

The identification of key journals and influential authors provides a roadmap for clinicians and researchers to access high-impact information. Journals such as Blood and Haematologica are instrumental in disseminating cutting-edge research findings, influencing clinical practice, and guiding future research directions. The prominence of certain authors and institutions highlights centers of excellence in PV research, which can serve as hubs for training, collaboration, and innovation. These hubs play a crucial role in driving forward the research agenda and ensuring the translation of scientific discoveries into clinical practice.

Recent research trends indicate a significant emphasis on the clinical implications of genetic findings, particularly concerning the development and evaluation of JAK2 inhibitors [22,23]. While these inhibitors represent a significant advancement in PV treatment, challenges such as drug resistance and side effects persist [23,24]. There is a growing need for research into combination therapies that can enhance efficacy and reduce adverse effects. Additionally, the focus on treatment outcomes and clinical trials underscores the importance of evidence-based practice in improving patient care. Long-term follow-up studies are essential to assess the durability of treatment responses and the potential long-term risks associated with new therapies. Prospective cohort studies and clinical trials with extended follow-up periods can be conducted to assess treatment responses and long-term outcomes in PV patients. Standardized outcome measures and rigorous monitoring protocols can be implemented to track disease progression and treatment-related adverse events. The expected outcome is an enhanced understanding of treatment efficacy and safety profiles over extended follow-up periods, along with the identification of optimal treatment strategies to improve long-term patient outcomes and quality of life.

**Figure 9:**
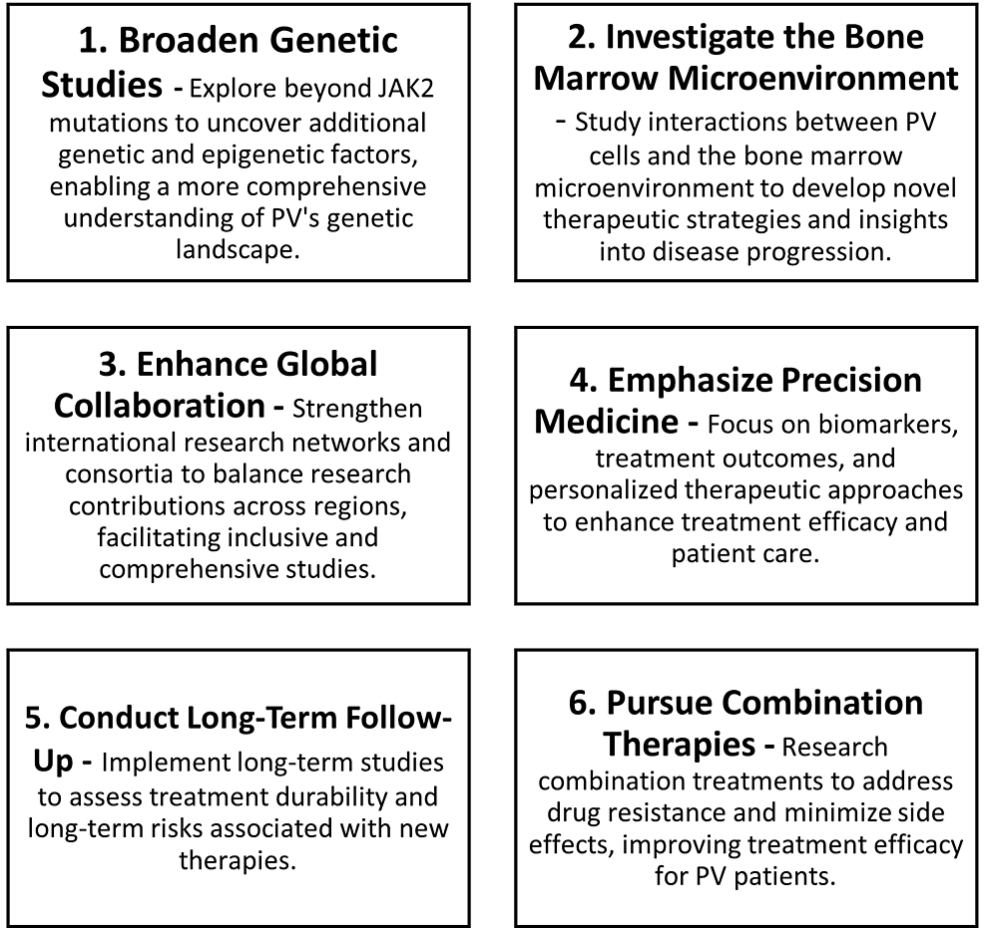
Future directions for PV research PV, polycythemia vera

Moreover, the shift toward precision medicine in PV research is evident, with a growing emphasis on identifying biomarkers for early diagnosis, disease monitoring, and treatment response. Integrating genomic, proteomic, and metabolomic data is crucial for developing comprehensive models that can guide personalized treatment strategies. Integrating precision medicine approaches into PV research and clinical practice can optimize diagnosis, treatment, and monitoring strategies. Precision medicine offers the potential to tailor interventions to individual patient characteristics, improving treatment efficacy and minimizing adverse effects. Comprehensive molecular profiling protocols can be developed to capture genomic, proteomic, and metabolomic data from PV patients. Machine learning algorithms and bioinformatics tools can be utilized to analyze multi-omics datasets and identify predictive biomarkers. The expected outcome is improved patient outcomes through personalized treatment regimens and targeted interventions, along with an enhanced understanding of disease heterogeneity and progression mechanisms, facilitating the development of more effective therapeutic strategies. However, the implementation of precision medicine requires significant investment in infrastructure, data management, and analytical tools, as well as addressing ethical and privacy concerns associated with genetic data. Figure 9 shows the future directions of PV research.

While significant progress has been made in PV research, particularly in understanding its genetic basis and developing targeted therapies, several challenges and gaps remain. Future research should broaden its scope to include other genetic and environmental factors, enhance global collaboration, and focus on precision medicine approaches. Addressing these challenges will require sustained effort, innovative research strategies, and a commitment to equitable global health. The dynamic and evolving landscape of PV research offers numerous opportunities for scientific inquiry and clinical innovation, ultimately aiming to improve the lives of patients affected by this chronic condition. These future directions reflect both the current trends in PV research as highlighted in recent literature and the gaps identified that warrant further investigation.

Implications

The bibliometric research on PV reveals critical implications for the scientific community and healthcare stakeholders. It highlights the increasing scientific interest in PV, emphasizing the necessity of addressing its complexities through global collaboration and strategic research initiatives. By identifying research trends, disparities among countries, and influential players in the field, the analysis offers valuable insights for policymakers, funding agencies, clinicians, and researchers. These insights can inform decisions regarding resource allocation, policy formulation, and research priorities, ultimately facilitating advancements in PV diagnosis, treatment, and patient care. Moreover, the identification of future research directions, including broadening genetic studies, investigating the bone marrow microenvironment, and emphasizing precision medicine, guides efforts to address remaining gaps in our understanding of PV and improve clinical outcomes.

Limitations

While bibliometric analysis provides valuable insights into the trends and patterns in PV research, it is important to acknowledge its limitations. One major limitation is its emphasis on the quantity of publications rather than the quality of the research. High publication counts do not necessarily correlate with high-impact or groundbreaking findings. Bibliometric metrics such as citation counts and h-index may favor well-established researchers and institutions, potentially overlooking innovative work from lesser-known sources. Additionally, bibliometric analysis often relies on databases that may not cover all relevant journals or include non-English language publications, leading to potential biases in the dataset. Finally, such analyses do not account for the context and nuances of research contributions, such as interdisciplinary collaborations or the practical application of findings in clinical practice. Recognizing these limitations is crucial for a balanced interpretation of the bibliometric data and for guiding future research directions in PV.

## Conclusions

This research paper provides a comprehensive analysis of PV research trends, highlighting significant advancements, key contributors, and emerging areas of focus. The findings underscore the increasing scientific and clinical interest in PV, as evidenced by a notable surge in research output over the past few decades. Prominent themes include the genetic basis of PV, advancements in targeted therapies such as JAK2 inhibitors, and the importance of precision medicine in improving patient outcomes. International collaboration plays a crucial role in advancing PV research, with diverse contributions from researchers and institutions worldwide. However, several gaps remain, including the need for broader genetic studies, exploration of the bone marrow microenvironment, and equitable distribution of research efforts across regions. The bibliometric research reveals critical implications for the scientific community and healthcare stakeholders by highlighting the necessity of addressing PV’s complexities through global collaboration and strategic research initiatives. These insights can inform decisions regarding resource allocation, policy formulation, and research priorities, ultimately facilitating advancements in PV diagnosis, treatment, and patient care. For clinicians, the emphasis on genetic studies and precision medicine suggests a shift toward more personalized treatment strategies, improving patient outcomes and quality of life. For researchers, understanding the importance of broadening genetic studies and investigating the bone marrow microenvironment can guide future research endeavors to fill existing gaps in knowledge. Policymakers and funding agencies can use these insights to allocate resources effectively, ensuring that high-impact research areas receive the necessary support. By addressing these challenges and embracing emerging opportunities, the field of PV research can continue to evolve, ultimately leading to improved diagnostics, treatments, and outcomes for patients affected by this chronic condition.
